# Probiotic VSL#3 Treatment Reduces Colonic Permeability and Abdominal Pain Symptoms in Patients With Irritable Bowel Syndrome

**DOI:** 10.3389/fpain.2021.691689

**Published:** 2021-09-22

**Authors:** Prapaporn Boonma, Jordan M. Shapiro, Emily B. Hollister, Shyam Badu, Qinglong Wu, Erica M. Weidler, Bincy P. Abraham, Sridevi Devaraj, Ruth Ann Luna, James Versalovic, Margaret M. Heitkemper, Tor C. Savidge, Robert J. Shulman

**Affiliations:** ^1^Department of Pathology, Texas Children's Microbiome Center, Texas Children's Hospital, Houston, TX, United States; ^2^Department of Pathology and Immunology, Baylor College of Medicine, Houston, TX, United States; ^3^Faculty of Medicine, King Mongkut's Institute of Technology Ladkrabang, Bangkok, Thailand; ^4^Department of Medicine, Baylor College of Medicine, Houston, TX, United States; ^5^Center for Pediatric Abdominal Pain Research, Texas Children's Hospital, Houston, TX, United States; ^6^Children's Nutrition Research Center, Houston, TX, United States; ^7^Department of Pediatrics, Baylor College of Medicine, Houston, TX, United States; ^8^Division of Gastroenterology, Houston Methodist Hospital, Houston, TX, United States; ^9^Department of Biobehavioral Nursing and Health Informatics, University of Washington, Seattle, WA, United States

**Keywords:** irritable bowel syndrome, VSL#3, probiotic, permeability, microbiome, bile acids

## Abstract

**Background:** Little is known regarding the clinical impact of treatment and treatment duration of probiotic VSL#3 on gut and microbiome function in irritable bowel syndrome (IBS). As part of a safety trial, we assessed the effect of VSL#3 treatment duration on abdominal pain, stooling, gut permeability, microbiome composition and function.

**Methods:** Adults with IBS were randomized into an open label trial to receive the probiotic VSL#3 for 4 or 8 weeks. Adverse events, abdominal pain, and stooling patterns were recorded daily. Gut permeability, fecal bile acid levels, and microbiome composition were profiled at baseline and after treatment.

**Results:** Fifteen subjects completed the trial (4-week: *n* = 8; 8-week: *n* = 7). Number of pain episodes decreased in both groups (*P* = 0.049 and *P* = 0.034; 4- vs. 8-week, respectively). Probiotic organisms contained in VSL#3 were detected in feces by whole shotgun metagenomic sequencing analysis and relative abundances of *Streptococcus thermophilus, Bifidobacterium animalis, Lactobacillus plantarum*, and *Lactobacillus casei* subsp. *paraccasei* correlated significantly with improved abdominal pain symptoms and colonic permeability at study completion. Although abdominal pain correlated significantly with the detection of probiotic species at study completion, a composite view of gut microbiome structure showed no changes in community diversity or composition after VSL#3 treatment.

**Conclusions:** Probiotic organisms identified in stool correlated significantly with improvement in colonic permeability and clinical symptoms, prompting future studies to investigate the mechanistic role of VSL#3 and colonic permeability in IBS pathophysiology in a larger randomized controlled trial.

**Clinical Trial Registration:** www.clinicaltrials.gov, Identifier: NCT00971711.

## Introduction

Irritable bowel syndrome (**IBS**) is common at all ages, from young children to adults, and the impact on quality of life can be significant (1–4). Understanding of the pathogenesis of IBS has evolved greatly over the past several decades. Once considered a disorder caused by abnormalities of gut smooth muscle, visceral hypersensitivity, and/or central nervous system hypervigilance, the past decade has seen a change in focus to include both central and peripheral mechanisms in the pathophysiology of IBS. Such peripheral mechanisms include abnormal colonic transit and rectal evacuation, intestinal intraluminal irritants, alterations in the gut microbiome, enteroendocrine cell dysfunction, low grade mucosal inflammation, and/or bile acid malabsorption (5, 6).

Alterations in gut microbiome community composition, as well as metabolites generated as a function of microbial metabolism (e.g., bile acids) have become ripe areas of study given the findings of alterations in some patients with IBS (7–10). These observations appear to have clinical relevance based on reports that probiotics can be efficacious as treatment for patients with IBS in both children and adults (7, 8, 11). Of these, there is evidence of efficacy based on randomized trials of VSL#3 in the treatment of IBS in children and adults (12–15).

The exact mechanism(s) whereby probiotics produce benefit in patients with IBS is still in question. Certain combination probiotics have been noted to alter gut microbial composition in humans, supporting the hypothesis that probiotics work by replacing or altering component bacteria of the gut (16, 17). In contrast, previous studies in humans using VSL#3 have shown disparate results with regard to its effects on gut microbiota composition, possibly related to differences in the duration of treatment or other factors (18–20). Similarly, some adults and children with IBS may have increased gut permeability which appears in some cases to relate to abdominal pain symptoms (21–23). To our knowledge, whether VSL#3 alters gut permeability in humans has not been studied.

In general, probiotics are thought to be safe except in the case of patients who are critically ill and/or immunocompromised (24). However, a recent review questions the presumed favorable risk/benefit ratio of treating IBS with probiotics (25). To our knowledge, no study of VSL#3 in adult patients with IBS has focused on potential side effects. In previous clinical trials of VSL#3 in IBS, it is not clear how adverse events were captured or reported (12–15, 19), although we previously reported no significant adverse events when the closely related probiotic VisBiome was administered to autistic children with functional gastrointestinal disorders (26).

We were requested by the United States Food and Drug Administration (FDA) to carry out a thorough safety study of VSL#3 in adults with IBS prior to its use in a randomized study in children with IBS. We made use of this opportunity to investigate in adult IBS patients with matched fecal omics data the effects of two dosing durations of VSL#3 on gut permeability, recovery of probiotic organisms and changes in gut microbiome composition determined by shotgun metagenomic sequencing, and metabolome (bile acid) profile alterations.

## Methods

### Study Design

The study was an investigator-initiated, phase I, open-label trial mandated by the FDA to assess the safety of VSL#3 in adults with IBS. Informed consent was obtained from the participants and the study was approved by the Baylor College of Medicine Institutional Review Board. The study was registered as clinicaltrials.gov identifier NCT00971711.

Patients were seen by a gastroenterologist (BA) who obtained a medical history and performed a physical exam to confirm the IBS diagnosis using Rome III criteria. Subjects who met entry criteria were randomized (1:1) to receive VSL#3 for either 4 or 8 weeks using a random number generator (www.randomization.com). Assignments were kept in sealed envelopes and were opened and recorded by the research pharmacy when dispensing the probiotic (Table 1).

**Table 1:** Schedule of study events.

**Action/event (days related to study drug)**	**Prior to**	**During/after**
	**study drug**	**study drug**
Gastroenterologist visit (history and physical exam)	−7	
Rome III questionnaire	−7	56 or 84
Informed consent	−7	
Medical exam	−14 or −7	
Pregnancy test	−2 to 0	
Stool microbiome/bile acid analysis	−7	28 or 56
GI permeability test	−7	28 or 56
VSL#3 administration		0-28 or 0-56
Pain/stooling diary	−7 to −1	0-42 or 0-70
AE and SAE diary	−7 to −1	0-42 or 0-70
Follow up call for symptoms and AE		56 or 84

A stool was collected for microbiome and bile acid analysis, and a GI permeability test was carried out prior to and after 4 or 8 weeks of probiotic administration. Weekly telephone calls were made to participants by the study staff to ensure compliance and assess any potential adverse events. While receiving the probiotic, participants kept a pain/stooling diary (Supplementary Table 1), recorded any concomitant medication use, and kept an adverse event (AE) and serious adverse event (SAE) diary (Supplementary Table 2).

A follow-up telephone call was made 1 month after completion of the probiotic treatment to inquire about any adverse events. To aid in assessment of compliance, participants were asked to return empty packets to study staff.

### Subjects

For recruitment, advertisements were placed in local papers and radio stations, and in gastroenterologists' offices. The consent form, along with the Rome III IBS module and the Functional Bowel Disorder Severity Index (27, 28), were mailed out to the participants to complete at home (Supplementary Table 1). Upon receipt of the consent form and the questionnaires by the investigators, the participants were screened further by telephone to assess eligibility. Inclusion/exclusion criteria listed in Supplementary Table 3 were applied. Subjects who passed screening were seen by an adult gastroenterologist (BA) as described above.

### Probiotic

The VSL#3 (Danisco®, Madison, WI) clinical trial product was obtained in December, 2011 and is made up of four strains of *Lactobacillus* (*L. casei* subsp. *paracasei, L. plantarum, L. acidophilus*, and *L. delbrueckii* subsp. *bulgaricus*), three strains of *Bifidobacterium* (*B. longum, B. infantis*, and *B. breve*), one strain of *Streptococcus thermophilus*, and starch. Although previously described as separate species, *B. infantis* and *B. longum* are now considered to be biotypes or subspecies of the same organism (*B. longum*). *Lactobacillus bulgaricus* in VSL#3 has been re-classified as *L. helveticus* (29, 30). Subjects took two packets daily (900 billion CFU) for either 4 or 8 weeks. The VSL#3 was mixed in a cold food or non-carbonated beverage.

### Stool Collection

A stool sample was collected prior to administration of the probiotic and on the final study day. Stools were collected in a self-sealing container placed over the toilet. Specimens then were placed in a freezer and transported by courier to the Texas Children's Microbiome Center and stored at −80°C until processed as previously described (31).

### Microbiome Analysis

Bacterial DNA was extracted using the PowerSoil DNA Isolation kit (MO BIO Laboratories, Carlsbad, CA, USA), with the Human Microbiome Project modifications to the manufacturer's protocol (32). Shotgun metagenomic sequence libraries (WGS) were generated and sequenced by the Human Genome Sequencing Center at Baylor College of Medicine (Houston, TX, USA). Each DNA sample was processed following a 101-bp, paired-end sequence library construction protocol with an average insert length of 200-bp, and was sequenced on the HiSeq2000 platform (Illumina Inc., San Diego, CA, USA). Raw sequence reads were quality filtered by VSEARCH (version 2.9) (33) using the options of—fastq_truncee of 1 and—fastq_minlen of 90. Taxonomic characterization of filtered reads was performed using the MetaPhlan2 package (34). Species profiles were used for alpha-diversity analysis using Shannon index, and was also used for beta-diversity analysis using principal coordinate analysis for abundance-weighted Jaccard distance profiling. ANOSIM test with 999 permutations was used to test the significance of sample groups, i.e., baseline vs. treated samples. Mann-Whitney tests with false discovery rate (Benjamini-Hochberg correction) were used to compare differences in the relative abundance of bacterial taxa including individual VSL#3 component species or the VSL#3 mixture as a whole between baseline and treatment groups. Spearman correlation analysis with Benjamini-Hochberg correction was performed when analyzing clinical variables with the relative abundance of individual VSL#3 component species.

### Permeability Test

Prior to starting the probiotic and at the end of the study treatment period, participants underwent a gastrointestinal permeability test (35). In brief, participants drank 100 mL of a solution containing sucrose (10 g), lactulose (5 g), mannitol (1 g), and swallowed a pill containing sucralose (1 g) followed by 240 ml of water after at least a 4-h fast after dinner. Subjects voided before drinking the solution and were told to avoid alcohol and non-steroidal antiinflammatory drugs 24 h prior to and during the permeability test. Each time the subject subsequently voided, urine was placed in separate containers. Thymol (33 μl of a 10% solution) was added as a preservative to each container provided to the participants. After the first morning void, participants were again allowed to eat and drink (with the exception of foods containing sucralose). Urine was collected for a total of 24 h starting from the time of ingestion of the sugars. The urine was placed in the freezer and transported by courier to Texas Children's Hospital after all urine had been collected. High-performance liquid chromatography was carried out to quantify the individual sugars as we and others have described (36). Percent sucrose recovery overnight was used to define gastric permeability and the lactulose/mannitol ratio and percent sucralose recovery over 24 h were used to define small intestinal and colonic permeability, respectively (37).

### Bile Acid Analysis

Stools from each treatment group were collected for bile acid analysis. Hot methanol bile acid extracts were analyzed by the Baylor College of Medicine Miraca Genetics Laboratory which provided quantitative measures of primary, secondary and conjugated bile acids [7α-hydroxy-4-cholestan-3-one—C4, HCO, cholic acid (CA), chenodeoxycholic acid (CDCA), glycocholic acid (GCA), taurocholic acid (TCA), glycochenodeoxycholic acid (GCDCA), taurochenodeoxycholic acid (TCDCA), deoxycholic acid (DCA), lithocholic acid (LCA), glycodeoxycholic acid (GDCA), taurodeoxycholic acid (TDCA). Additional bile acids measured included: ursodeoxycholic acid (UDCA, ursodiol) and tauroursodeoxycholic acid (TUDCA)]. Bile acid concentrations (ng/ml) were correlated with clinical symptoms, intestinal permeability and microbiome features as described in the statistics section.

### Pain and Stool Diaries

Subjects kept diaries of pain episodes, stools, and adverse events during a 1-week baseline period, during VSL#3 administration, and for 2 weeks after completion of probiotic administration. The data was called to a 24-h phone line as previously described (38). Subjects recorded all pain episodes they experienced in a day, documenting time, duration, and body location. Subjects also reported all stools in a day, noting whether or not they had a stool, if there was pain associated with stooling, and the type of stool as described by the Bristol Stool Scale (39). Thus, participants in the 4-week group recorded up to 49 days of pain episodes and stools, and those in the 8-week group up to 77 days.

### Adverse Events

Adverse events were recorded daily using Supplementary Table 2. Subjects were called by study coordinators weekly for 4 or 8 weeks to assess any adverse events which may have occurred during the prior week, while taking, and for 2 weeks after completion of VSL#3. Follow-up at 1 month after study completion occurred via telephone call from the study coordinator. As per the FDA, adverse events were defined as “any untoward medical occurrence in a patient or clinical investigation subject administered a pharmaceutical product and which does not necessarily have to have a causal relationship with this treatment.”

### General Statistics

Statistics were analyzed using SPSS® 23 (IBM Corporation, Armonk, NY). Total number of pain episodes over the study periods, mean pain rating over the study periods, and mean of the maximum ratings of pain per day over the study periods were compared using Mann-Whitney *U*-tests. Assessment of pain data in the 4- and 8-week groups combined was carried out using Wilcoxon Signed Ranks tests. Number of bowel movements, number of days with no bowel movement, and median stool type were compared using Mann-Whitney *U*-tests. Data are shown as mean ± SD. Pearson and Spearman correlations between probiotic and microbial species, bile acids concentrations, abdominal pain frequency and intestinal permeability in each treatment group were conducted. Benjamini-Hochberg false discovery rate correction was applied to compare microbiome correlations and group differences.

## Results

**Figure 1:**
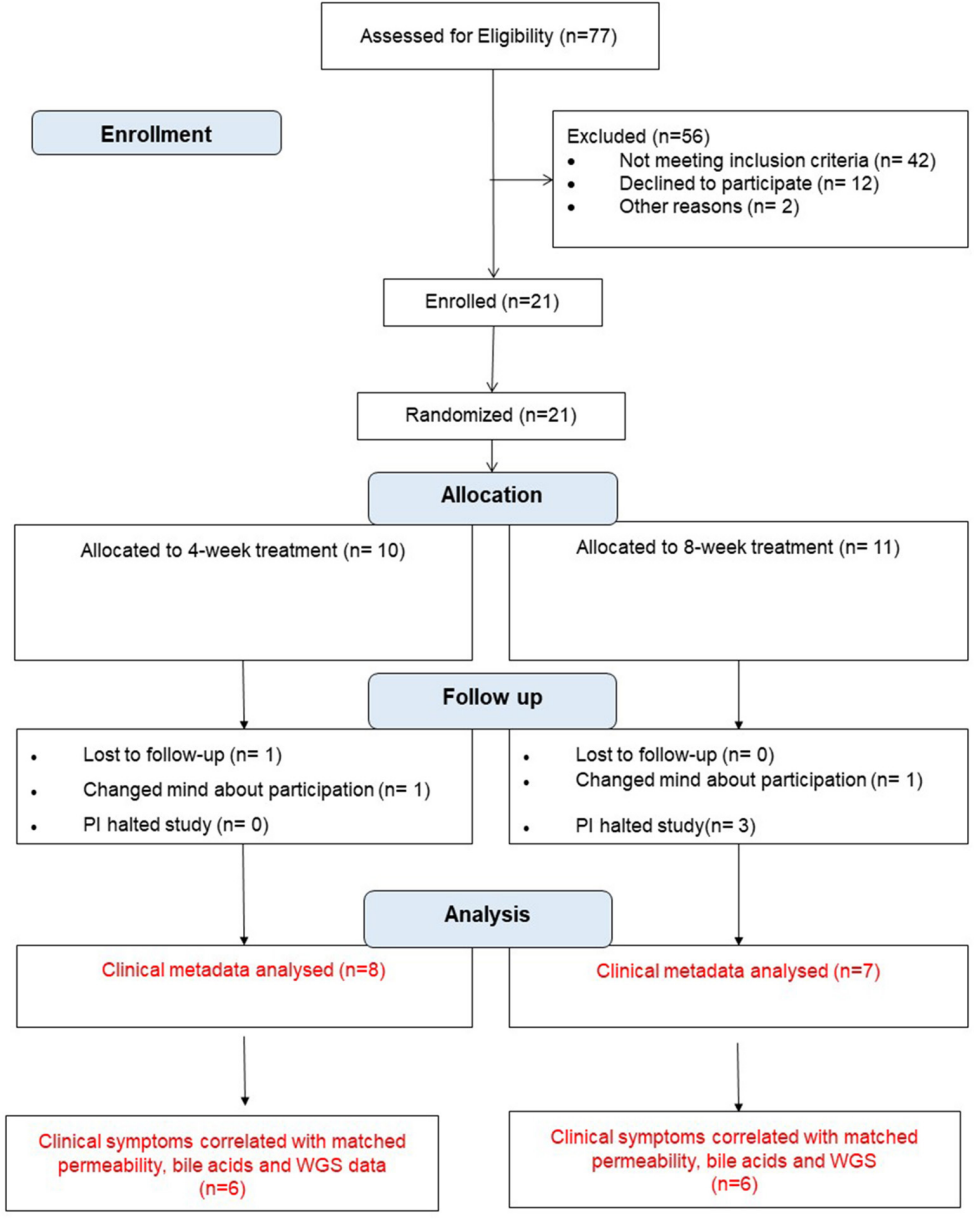
Consort diagram of probiotic VSL#3 treatment in patients with irritable bowel syndrome.

Of the 77 individuals screened, 21 were enrolled and provided product safety data (Figure 1). Demographic and clinical characteristics of the participants are presented in Table 2. The first two participants did not provide urine or stool samples as these collections were added to the study design after the trial began. Three participants dropped out of the study. Three participants were halted on the study when it was discovered that the research pharmacy had dispensed expired VSL#3 to them. Parenthetically, samples of the expired product were sent back to the manufacturer (Danisco) for analysis which revealed that the product still contained the appropriate colony forming units (CFU) of bacteria (i.e., matched the Certificate of Analysis). Therefore, permission was obtained from the FDA to continue the study. Recruitment was carried out between January 2011 and January 2014.

**Table 2:** Demographic and clinical characteristics of 4- and 8-weeks treatment group.

	**4-week treatment group**	**8-week treatment group**
	**(*n* = 8)**	**(*n* = 7)**
**Demographic and clinical characteristics**		
Age (years)	36 ± 10	36 ± 9
**Gender**		
Male	2	2
Female	6	5
**Ethnicity**		
Non-hispanic	7	6
Hispanic	1	1
**Race**		
Caucasian	3	3
African American	3	3
Asian	1	0
Hispanic	1	1
BMI	25 ± 5.3	25 ± 5.4
IBS severity score	249.8 ± 85.5	242.6 ± 66.9
Starting pain severity	37.4 ± 24.1	42.4 ± 20.3
**IBS subtype**		
Constipation	2	2
Diarrhea	0	1
Mixed	6	4

*Mean ± SD.

### Pain and Stool Diaries

Total number of pain episodes, mean pain rating over the study periods, and mean of the maximum ratings of pain per day over the study periods pre- and post-VSL#3 administration were not different between the 4- and 8-week groups (Table 3). However, assessing 4- and 8-week groups together, there were significant decreases in the number of pain episodes and mean of the maximum pain rating after VSL#3 administration as compared to before probiotic (*P* = 0.007 and *P* = 0.004, respectively). Assessed individually, the number of pain episodes was significantly decreased after VSL#3 administration in both 4- and 8-week groups (*P* = 0.049 and *P* = 0.034, respectively). Additionally, when assessed individually, the 8-week group had a decrease in the mean of the maximum pain rating after VSL#3 administration (*P* = 0.018). The number of bowel movements per day, number of days without a bowel movement, and mean stool type did not differ between groups (Table 3).

**Table 3:** Pain and stooling results of 4- and 8-weeks group.

**Parameter**	**4-week group**		**8-week group**	
	**Baseline**	**Post-treatment**	**Baseline**	**Post-treatment**
**Pain and stooling results**				
**Pain**				
Number of pain episodes	6.8 ± 5.0^‡^	2.0 ± 2.4^#^	6.4 ± 4.2	2.0 ± 2.7^#^
**Mean pain rating**				
Mean of maximum pain ratings	3.8 ± 1.7	2.1 ± 2.4*	3.9 ± 2.2	1.5 ± 1.8*
**Stooling**				
Number of bowel movements per day	1.5 ± 0.6	1.4 ± 0.9	1.1 ± 0.8	1.3 ± 0.9
Number of days with no bowel movement	1.3 ± 1.3	1.1 ± 1.4	1.9 ± 2.4	1.8 ± 2.8
Median bristol stool scale type	3.9 ± 1.6	4.3 ± 1.3	3.2 ± 1.9	3.4 ± 1.4

*‡Mean ± SD.*

*P = 0.049 for post-treatment change within each group.

*#P = 0.034 for post-treatment change within each group*.

### Microbiome Analysis of the Pure VSL#3 Product

Of the 15 study participants who completed the probiotic trial with clinical metadata, only 12 subjects provided sufficient fecal material to facilitate matched deep shotgun metagenomic sequencing (WGS) and bile acid profiling. WGS analysis was performed using MetaPhlan2 to positively identify the presence of VSL#3 containing *L. casei* subsp. *paracasei* (38.93%), *L. helveticus* (2.45%), *L. plantarum* (1.20%), *L. acidophilus* (1.36%), *S. thermophilus* (32.89%), *B. animalis* (11.14%), and *B. breve* (11.33%). The abundance of *B. longum* listed as a probiotic species in VSL#3 was 0.00148% indicating that this organism is barely detected.

### Microbiome Analysis–Whole Genome Shotgun Sequencing

**Figure 2:**
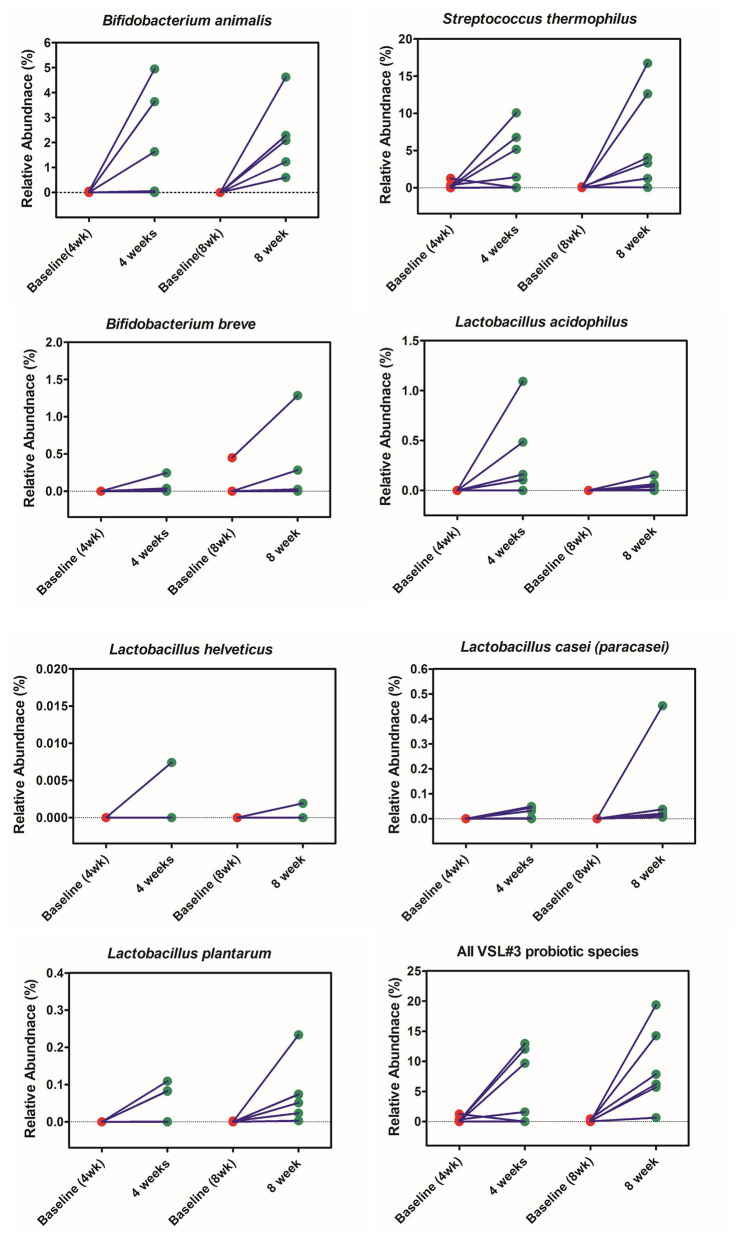
Relative abundance of organisms found in VSL#3 in stool after both 4- and 8-weeks of treatment.

**Figure 3:**
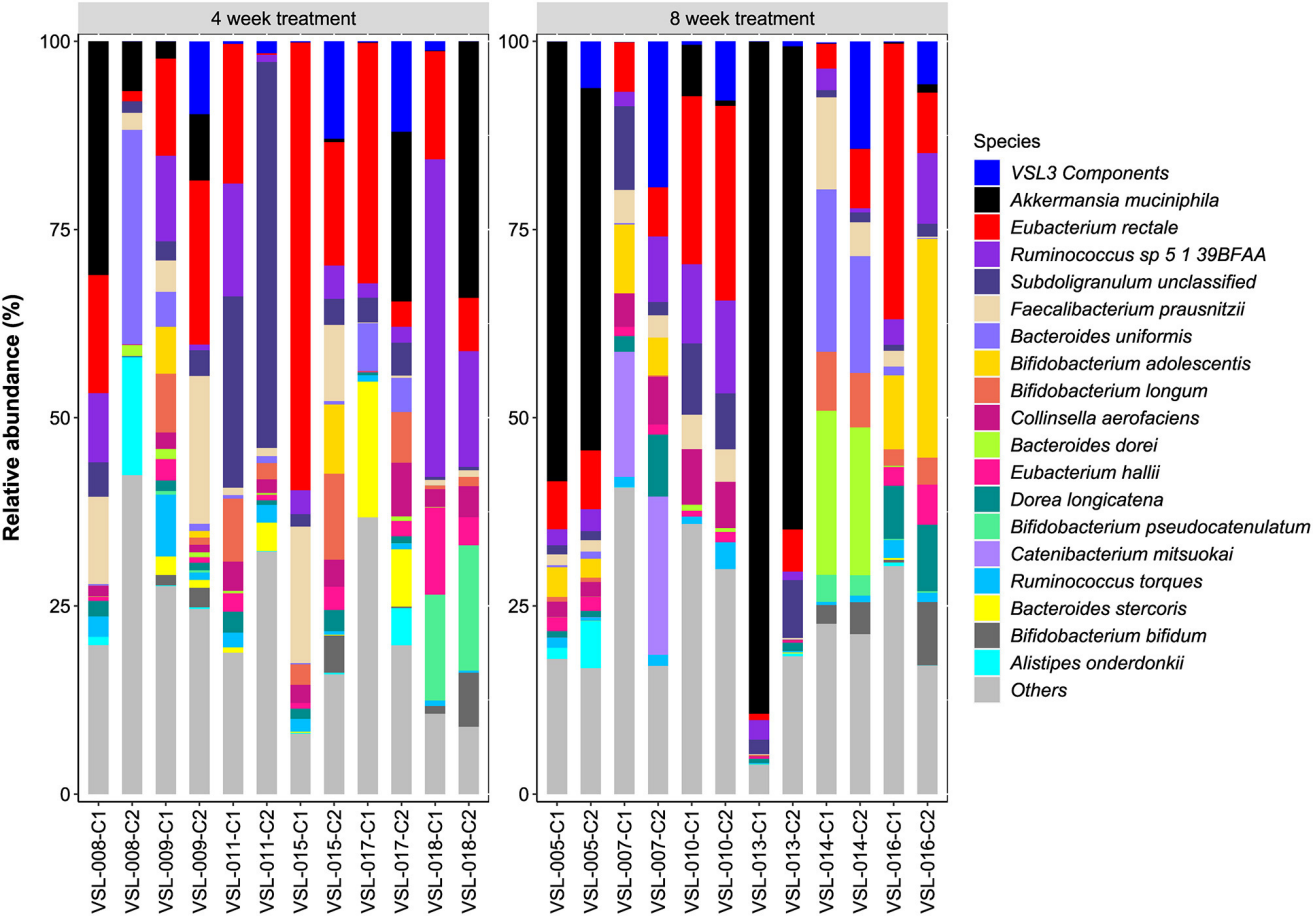
Species-level relative abundances of community members after both 4- and 8-weeks of treatment.

The probiotic species found in VSL#3 (guided by our WGS analysis of the ingested pure product) increased in relative abundance in stool after both 4- and 8-weeks of treatment (Figure 2). The relative abundance of individual VSL#3 species was not affected by treatment duration but when the two treatment groups were considered together to facilitate statistical power, significant enrichment of *B. breve, B. animalis, L. plantarum, L. casei, L. acidophilus*, and *S. thermophilus* was observed (*P* < 0.05 for each; Mann-Whitney Rank test with FDR correction). Of note, *B. animalis* and *S. thermophilus* were highly enriched after probiotic administration, whereas Lactobacillus species were detected at lower relative abundance after either 4 or 8 weeks. The relative abundance of several microbiome community members were altered following VSL#3 consumption, although these shifts were not consistent enough across subjects to be statistically significant (Figure 3). As recently reported (40), unique host and microbiota-dependent factors determine empiric probiotic colonization in humans and analysis of individual subjects in this study supported this corresponding microbiota heterogeneity.

**Figure 4:**
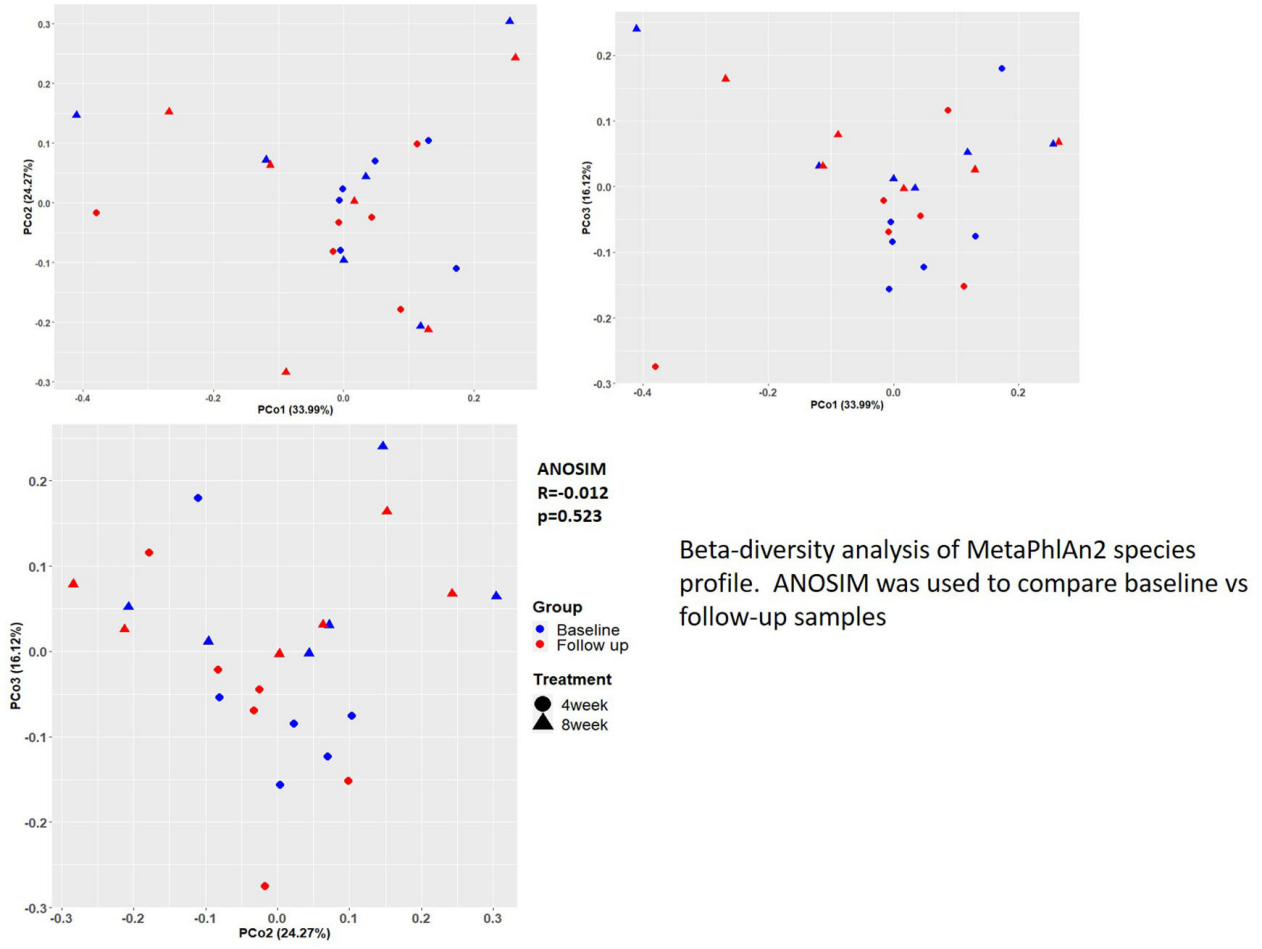
Beta-diversity analysis of MetaPhlAn2 species profile. ANOSIM was used to compare baseline vs. follow-up samples.

Although significant changes were found for several VSL#3-related species, beta-diversity analysis of MetaPhlAn2 species profile supported the notion that probiotic administration did not significantly alter fecal microbiota community diversity (Figure 4). Spearman correlations measured for identified VSL#3 species abundances of *L. plantarum, L. casei* subsp. *paraccasei, B. animalis*, and *S. thermophilus* with the number of pain episodes were rho = −0.59 (*p* = 0.003, BH corrected *p* = 0.030), rho = −0.67 (*p* = 0.0004, BH corrected *p* = 0.010), rho = −0.64 (*p* = 0.001, BH corrected *p* = 0.018) and rho = −0.34 (*p* = 0.118, BH corrected *p* = 0.42), respectively, demonstrating significant inverse correlations between probiotic species abundance in feces with reported pain frequency.

### Gastrointestinal Permeability

**Figure 5:**
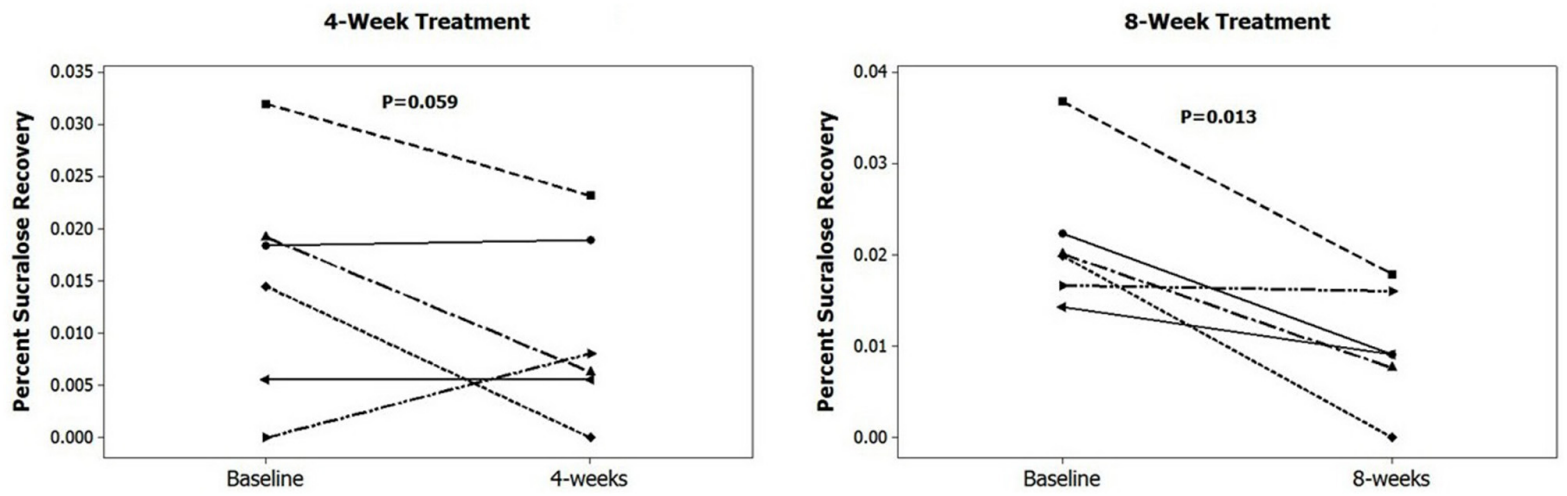
Colonic permeability (percent sucralose recovery) of probiotic VSL#3 treatment in 4- and 8-weeks group.

There was a trend for colonic permeability (percent sucralose recovery) to correlate positively with number of pain episodes but statistical significance was not achieved (rho = 0.36; raw *p* = 0.09). Similarly, colonic permeability decreased with VSL#3 probiotic treatment duration and reached statistical significance after 8-weeks of probiotic treatment (Figure 5). These trends need to be validated in larger randomized trials in order to explore possible disease mechanism.

### Bile Acids

Bile acid profiles did not change significantly between baseline and after probiotic treatment when applying multiple testing corrections (data not shown), and require a sufficiently powered trial to explore possible trends that we identified. In particular, unconjugated secondary bile acids associated negatively with colonic permeability (rho = −0.39; raw *p* = 0.064, BH corrected *p* = 0.491), whereas, primary bile acids associated positively with stool type (rho = 0.48; raw *p* = 0.020, BH corrected *p* = 0.550).

### Adverse Events

Safety data included all 21 subjects. No deaths, infections with component bacteria, unscheduled IBS-related health care visits, change in frequency or severity of events, or reported causality occurred in subjects while taking VSL#3.

There were 88 AEs reported (Supplementary Table 4); all were rated as not serious according to the FDA rating scale. The AEs were deemed not related to VSL#3 as the symptoms reported as AEs are common symptoms in IBS. Bloating was the most common AE reported followed by having ≥ 4 stools in a 24-h period.

### Serious Adverse Events

Two SAEs were reported during the study in the same subject who was admitted to the hospital for vaginal bleeding due to uterine fibroids and required administration of blood products. These SAEs, the first of which occurred while the subject was on VSL#3, were deemed to be unrelated to the probiotic.

## Discussion

Many gaps still remain in our understanding of how probiotics impact the gut microbiome in patients with IBS. Our study aimed to comprehensively assess the pre- and post-VSL#3 gut response at the level of the microbiome using deep resolution WGS, metabolome (bile acids), and the gut barrier integrity (permeability).

VSL#3 has been shown previously to be effective in improving symptoms in adults and children with IBS in double blind, randomized trials (12–14). However, an understanding of the mechanism(s) responsible remain elusive. The response of the microbiome to probiotics seems to hold promise in helping to understand the mechanisms by which gut microbiota contribute to symptoms in IBS. Only one previous study, to our knowledge, examined the gut microbiome response to VSL#3 administration. Michail et al. did not find the gut microbiome to be altered after 8 weeks of treatment (19). However, their study employed microarray technology which potentially could limit their ability to detect changes (19). In our own study of Visbiome—a closely related probiotic mixture to VSL#3—we found significant improvement in the quality of life score (PedQL) in autistic children that correlated with detectable Lactobacillus spp abundance in fecal specimens (26). However, this 16S rDNA amplicon-based study lacked the species resolution provided by the WGS sequencing employed in the current study to identify specific probiotic strains associated with these health benefits. Although our WGS analysis did not identify robust VSL#3-induced compositional changes in the gut microbiome of IBS patients, probiotic species *L. plantarum, L. casei subsp. paraccasei, B. animalis*, and *S. thermophilus* were found to correlate significantly with number of pain episodes. These probiotic species also were associated with trends in reduced colonic permeability, although a larger randomized trial of IBS patients validating probiotic correlations with colonic permeability and abdominal pain symptoms is required. It is not clear how permeability is linked mechanistically with abdominal pain (21), but this could relate to direct microbial-regulation of the epithelial barrier, allowing access to submucosal immune cells which in turn can activate sensory nerves directly (41, 42). Probiotic and microbiome species also have the potential to directly relay nociceptive signals to sensory intrinsic afferent neurons in the intestine.

To our knowledge, gut permeability has not been measured following VSL#3 administration to adults with IBS. Animal models of gut inflammation have suggested that administration of VSL#3 is associated with decreased gut permeability, perhaps acting through increases in the expression of phosphorylated p38 and ERK and/or preventing apoptosis and maintaining tight junction protein expression (43, 44). A number of studies have shown that increased gut permeability can be related to abdominal pain symptoms in IBS (45). Whether the improvement in IBS symptoms reported with VSL#3 and other probiotics relates directly to normalization of permeability awaits further studies.

Several of the VSL#3 species identified in the fecal specimens have the potential to transform bile acids. It is possible that altered metabolic activity of the microbiota leads to an imbalance in fecal bile acid profiles in IBS cases, as we and others recently have reported (46). Although our study was underpowered to make statistical claims, we found trends that merit further investigation in larger trials indicating that taurochenodeoxycholic acid is associated with increased abdominal pain and colonic permeability, which could reflect a direct bile acid interaction on epithelial tight junction activity. In a prior omics study of IBS children we observed a similar abdominal pain association with taurochenodeoxycholic acid (47), and deconjugation by VSL#3 could constitute a protective mechanism as this is a bile acid species that is also known to disrupt epithelial barrier integrity.

From the abundance profile of individual patients at species level, we found that the sum of the relative abundances of probiotic species increased after 4 or 8 weeks of VSL#3 administration. Specifically, *B. animalis* and *S. thermophilus* were more abundant than the other VSL#3-related species and both demonstrated negative correlations with pain frequency, although the latter organism did not meet statistical rigor after multiple-testing correction. Although, *S. thermophilus* has not been associated previously with pain resolution in clinical trials, one previous clinical trial demonstrated *B. animalis* DN-173 to be associated with improved health-related quality of life and abdominal pain in adults IBS patients (46). Several other clinical studies also indicate that *B. longum*, which is not detected in VSL#3 product used in this study, is also a potential species for pain management in IBS patients (48).

The primary bile acids (CA and CDCA) are synthesized from cholesterol in the liver and are then released and transported to the small intestine, where a minority amount enters the large intestine for conversion to secondary bile acids by the gut microbiota. Bile acids play a role in IBS pathophysiology and in some IBS patients abdominal pain is linked with bile acid malabsorption (44, 45, 49). Lactobacilli and Bifidobacteria subspecies are able to de-conjugate and absorb bile acids, as well as perform bile acid biotransformation which may detoxify species that are linked with increased intestinal permeability and pain (50). Dior et al., demonstrated bile acid deconjugation activity was decreased in IBS (51). Although we observed trends in bile acid profiles that support a potential pathogenic role in modulating abdominal pain and colonic permeability, these results did not meet the statistical rigor to establish precedent for bile-acid mediated protection by VSL#3.

In summary, our results show that 4-week treatment with VSL#3 is as effective as 8-weeks in populating the gut with probiotic organisms. Amelioration of abdominal pain correlated with abundance of probiotic organisms detected in study participants, indicating that future studies should focus on identifying probiotic signals to the host. We have previously reported in a constipated animal model that *Lactobacillus* probiotic retention is critical in communicating effectively with the host, in this case *via* modulation of visceral opioid receptors (52). Our study had some limitations, given the relatively small sample size and the open trial design. Strengths of our study included the deep-resolution metagenomics used for probiotic and structural microbiome characterization, with simultaneous physiological measurement of gut permeability and bile acid profiles. The permeability results would not have been affected by the open label nature of our study, and sets the stage for a future double blind randomized controlled trial to control for significant placebo effects observed in IBS patients.

## Data Availability Statement

The datasets presented in this study can be found at https://www.ncbi.nlm.nih.gov/bioproject/PRJNA725223.

## Ethics Statement

The studies involving human participants were reviewed and approved by Baylor College of Medicine Institutional Review Board. The study was registered as clinicaltrials.gov identifier NCT00971711. The patients/participants provided their written informed consent to participate in this study.

## Author Contributions

All authors listed have made a substantial, direct and intellectual contribution to the work, and approved it for publication.

## Funding

This study was supported in part by R01 NR05337 and R34 AT006986, U01-AI24290, P01-AI152999 R01-AI10091401, UH2DK093990 and UH3DK083990 from the National Institutes of Health, the Daffy's Foundation, the USDA/ARS under Cooperative Agreement No. 58-3092-0-001, and P30 DK56338 which funds the Texas Medical Center Digestive Disease Center.

## Author Disclaimer

The content is solely the responsibility of the authors and does not necessarily represent the official views of the National Institutes of Health. This work is a publication of the USDA/ARS Children's Nutrition Research Center, Department of Pediatrics, Baylor College of Medicine and Texas Children's Hospital. The contents do not necessarily reflect the views or policies of the USDA, nor does mention of trade names, commercial products, or organizations imply endorsement by the US Government.

## Conflict of Interest

The authors declare that the research was conducted in the absence of any commercial or financial relationships that could be construed as a potential conflict of interest.

## Publisher's Note

All claims expressed in this article are solely those of the authors and do not necessarily represent those of their affiliated organizations, or those of the publisher, the editors and the reviewers. Any product that may be evaluated in this article, or claim that may be made by its manufacturer, is not guaranteed or endorsed by the publisher.
