# Supplementary material for: Probiotic VSL#3 Treatment Reduces Colonic Permeability and Abdominal Pain Symptoms in Patients With Irritable Bowel Syndrome
Source: Front Pain Res (Lausanne). 2021 Sep 22;2:691689. doi: 10.3389/fpain.2021.691689 (PMC8915646; doi:10.3389/fpain.2021.691689)

**Supplemental Table 1**

Subject ID: __________________ Date: _____________

Day #: ____________

**Daily Diary or Any Pain Episodes**

**IF** you had pain today, draw a vertical line across the horizontal line to show the severity of the pain (as was explained to you previously). If you did not have pain, **DO NOT** draw a line but circle “No pain at all.”

**No pain at all _________________________________________ The worst pain you can imagine**

**IF** you had pain today, record the time and how long it lasted:

Time _________ AM PM Duration______________

**IF** you had more than one pain episode today, record them below:

**No pain at all _________________________________________ The worst pain you can imagine**

Time _________ AM PM Duration______________

**No pain at all _________________________________________ The worst pain you can imagine**

Time _________ AM PM Duration______________

**No pain at all _________________________________________ The worst pain you can imagine**

Time _________ AM PM Duration______________

**No pain at all _________________________________________ The worst pain you can imagine**

Time _________ AM PM Duration______________

**
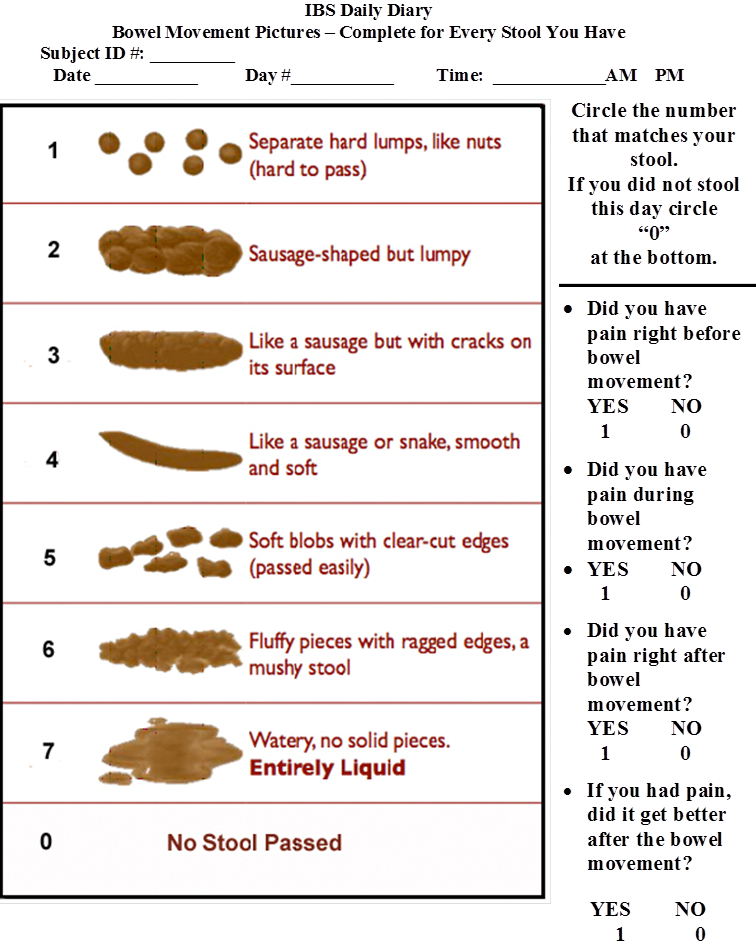
**

**Pain Location - Complete Every Time You Have Pain**

**Subject ID #: _________**

**Date: __________________**

**Day #___________**

**Time: ____________AM PM**


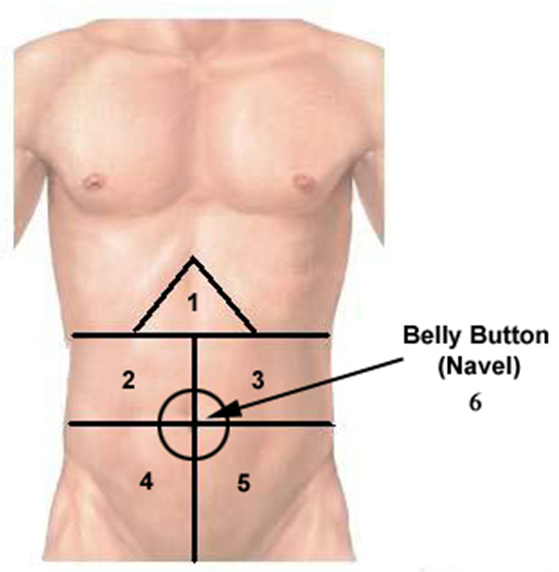

Supplement: Supplementary file 1 [file Table_1.docx]
