# Supplementary material for: Probiotic VSL#3 Treatment Reduces Colonic Permeability and Abdominal Pain Symptoms in Patients With Irritable Bowel Syndrome
Source: Front Pain Res (Lausanne). 2021 Sep 22;2:691689. doi: 10.3389/fpain.2021.691689 (PMC8915646; doi:10.3389/fpain.2021.691689)
Supplement: Supplementary file 2 [file Table_2.docx]

**Supplemental Table 2**

Subject ID: __________________ Date: _____________

Day #: ____________

**Daily Diary for Possible Adverse Events**

| ***Please complete the following Table every day*** | |
| --- | --- |
| Did you have 4 or more stools in 24 hours? | No Yes* |
| Did you vomit 3 or more times in 24 hours? | No Yes* |
| Total Number of times you vomited |  |
| Did you have constipated that interfered with your activity? | No Yes* |
| Write in your temperature for today | ° F |
| Was your temperature 100.4 ° F or more? | No Yes* |
| Did you have chills? | No Yes* |
| Did you have bloating? | No Yes |
| Did you have a bloody stool? | No Yes* |
| Did you call your doctor today because of your abdominal pain? | No Yes* |
| IF you called your doctor did he/she give you a new medicine for the abdominal pain? | No Yes* |
| IF you called your doctor did he/she change the dose of medicine you already were on because of the abdominal pain? | No Yes* |
| Did you visit the emergency room or were you hospitalized? | No Yes* |

- If you answered YES to any of the questions with an asterisk (*) please call Dr. Robert J. Shulman at XXX and Dr. Bincy Abraham at XXX.
- Call Dr. Robert J. Shulman at XXX and Dr. Bincy Abraham at XXX if you have chills, temperature of 100.4ºF or more, if you called your doctor or otherwise sought medical attention for any symptom, if your doctor prescribed a new medication for the abdominal pain or changed the dose of the medication you were taking for abdominal pain, if you vomited three or more times in 24 hours or if vomiting interfered with your activity, if you had four or more stools in 24 hours, if you have had constipation that interfered with activity, if you have had no stool for a week, have had bloody stool, if you have sought medical attention for any reason, visited the emergency room (ER), or been hospitalized.
