# Supplementary material for: Probiotic VSL#3 Treatment Reduces Colonic Permeability and Abdominal Pain Symptoms in Patients With Irritable Bowel Syndrome
Source: Front Pain Res (Lausanne). 2021 Sep 22;2:691689. doi: 10.3389/fpain.2021.691689 (PMC8915646; doi:10.3389/fpain.2021.691689)
Supplement: Supplementary file 3 [file Table_3.docx]

**Supplemental Table 3**

**Inclusion and Exclusion Criteria**

| **Inclusion Criteria** |
| --- |
| 1. 18 years of age and up to 60 years of age |
| 1. Otherwise well and meet the criteria for IBS as defined by the Rome III criteria as noted above |
| 1. Medical evaluation reveals no organic reason for the abdominal pain |
| 1. The subject does not have a serious chronic medical condition (e.g., diabetes) |
| 1. Ability to speak and understand English |
| 1. Telephone access |
| 1. IBS Severity Scale score of ≥ 75 |
| **Exclusion Criteria** |
| 1. Organic disease accounting for GI symptoms |
| 1. Chronic illness such as renal disease, congenital heart disease, diabetes, moderate or severe asthma, abdominal surgery, or immunosuppressed (e.g., organ transplant recipient) |
| 1. Have received extraneous probiotic (i.e., not in a food such as yogurt) within 4 months of starting the study |
| 1. Subjects who are taking prescription or over-the-counter medications for GI disorders that completely relieve their symptoms because by definition these individuals do not have IBS (e.g., antacids, proton pump inhibitors, histamine receptor antagonists) |
| 1. Medication allergies or contraindications which would preclude antimicrobial treatment for potential infection with VSL#3 component organisms |
| 1. Pregnancy |
| 1. Subjects who have an individual in the household who is immunosuppressed (e.g., genetic immune disorder, organ transplant) |
| 1. Oral temperature ≥ 38.0ºC |
| 1. Poorly controlled hypertension, history of cardiac disease, stroke/CVA, bowel ischemia, or other risk factors for bowel ischemia |
| 1. History of acute or chronic pancreatitis |
| 1. Cardiac valvular disease or other risk factor for endocarditis |
| 1. Subjects who indicate on the IBS scoring questionnaire that their pain is “severe” or “very severe.” |
| 1. Subjects who pain lasts more than 5 out of 10 days. |
| 1. Subjects whose scores indicate more than mild IBS who are over 45 years of age |
