# Supplementary material for: Probiotic VSL#3 Treatment Reduces Colonic Permeability and Abdominal Pain Symptoms in Patients With Irritable Bowel Syndrome
Source: Front Pain Res (Lausanne). 2021 Sep 22;2:691689. doi: 10.3389/fpain.2021.691689 (PMC8915646; doi:10.3389/fpain.2021.691689)
Supplement: Supplementary file 4 [file Table_4.docx]

**Supplemental Table 4**

**Adverse Events**

| **Adverse Events** | **Number of Episodes** | **# of Subjects Reporting AE** |
| --- | --- | --- |
| Bloating | 54 | 12 |
| ≥ 4 stools/24 hr | 16 | 5 |
| Constipation | 6 | 1 |
| Abdominal pain | 5 | 2 |
| Bloody stool | 3 | 3 |
| Hospitalization | 2 | 1 |
| Nausea | 1 | 1 |
| Chills | 1 | 1 |
| **Total AEs** | 88 |  |
| **Subjects With No AEs** | 3 | 3 |
| **Subjects With Unknown AEs** | 1 | 1 |
